# Analysis of optimal phenotypic space using elementary modes as applied to *Corynebacterium glutamicum*

**DOI:** 10.1186/1471-2105-7-445

**Published:** 2006-10-12

**Authors:** Kalyan Gayen, KV Venkatesh

**Affiliations:** 1 Department of Chemical Engineering, Indian Institute of Technology, Bombay, Powai, Mumbai-400 076, India; 2 School of BioSciences & Bioengineering, Indian Institute of Technology, Bombay, Powai, Mumbai-400 076, India

## Abstract

**Background:**

Quantification of the metabolic network of an organism offers insights into possible ways of developing mutant strain for better productivity of an extracellular metabolite. The first step in this quantification is the enumeration of stoichiometries of all reactions occurring in a metabolic network. The structural details of the network in combination with experimentally observed accumulation rates of external metabolites can yield flux distribution at steady state. One such methodology for quantification is the use of elementary modes, which are minimal set of enzymes connecting external metabolites. Here, we have used a linear objective function subject to elementary modes as constraint to determine the fluxes in the metabolic network of *Corynebacterium glutamicum*. The feasible phenotypic space was evaluated at various combinations of oxygen and ammonia uptake rates.

**Results:**

Quantification of the fluxes of the elementary modes in the metabolism of *C. glutamicum *was formulated as linear programming. The analysis demonstrated that the solution was dependent on the criteria of objective function when less than four accumulation rates of the external metabolites were considered. The analysis yielded feasible ranges of fluxes of elementary modes that satisfy the experimental accumulation rates. In *C. glutamicum*, the elementary modes relating to biomass synthesis through glycolysis and TCA cycle were predominantly operational in the initial growth phase. At a later time, the elementary modes contributing to lysine synthesis became active. The oxygen and ammonia uptake rates were shown to be bounded in the phenotypic space due to the stoichiometric constraint of the elementary modes.

**Conclusion:**

We have demonstrated the use of elementary modes and the linear programming to quantify a metabolic network. We have used the methodology to quantify the network of *C. glutamicum*, which evaluates the set of operational elementary modes at different phases of fermentation. The methodology was also used to determine the feasible solution space for a given set of substrate uptake rates under specific optimization criteria. Such an approach can be used to determine the optimality of the accumulation rates of any metabolite in a given network.

## Background

Application of metabolic engineering towards strain improvement involves detailed quantitative evaluation of cellular physiology [1-3]. Determination of intracellular metabolic fluxes helps in gaining valuable insights into the functioning of the active cellular metabolism, the knowledge of which aids in the development of rational strategies for strain improvement [4]. Theoretical methods have been developed for predicting key aspects of network functionality for a given metabolic network [5-8]. Experimental methods in tandem with theoretical analysis are key strategies for successful application of metabolic engineering to optimize the productivity of a native strain [9].

Most theoretical methods, such as metabolic flux analysis (MFA), are based on stoichiometric reactions involving various metabolites in a metabolic network [10-13]. The reaction network is used in conjunction with measured accumulation rates of certain metabolites as a constraint to determine the fluxes. Linear programming is used to maximize an objective function in the presence of stoichiometric constraints, which is also used in flux balance analysis (FBA) [7,14]. These methods have gained popularity among many researchers as seen through its application to various microbial systems. Recently, the reaction details in a metabolic network have been used to determine elementary modes, which are minimal set of enzymes connecting the external metabolites [6,15,16]. Certain advantages have been associated with analysis involving elementary modes such as ease in evaluating maximum yields of metabolites and the flux distribution inherently ensuring the directionality of reactions. Elementary modes have also been used to determine the fluxes of a metabolic network using matrix algebra [17]. However, both the methodologies (i.e. FBA and elementary modes analysis) require experimentally determined rates. Typically, the measurements of the extracellular metabolites are used in the analysis assuming pseudo steady state levels of the intracellular metabolites. It is relevant to raise the question regarding the minimum number of accumulation rates of extracellular metabolites obtained through experiments, which are necessary for proper assessment of fluxes in a network. We address this issue by analyzing the flux distribution of *Corynebacterium glutamicum *for the production of amino acids (lysine) using elementary modes.

Flux distribution in the metabolic network of *C. glutamicum*, which is used for lysine production, is well demonstrated. Batch growth of *C. glutamicum *for lysine production can be represented through four phases. Phase-I represents balanced growth with little or no product formation and is dependent on threonine concentration. Phase-II represents high lysine synthesis and biomass production rate with constant respiration rate. In phase-III, lysine production continues at a high rate while biomass productions saturates with a decrease in respiration rate. Phase-IV sees a gradual reduction in lysine synthesis and redirection of lysine to other byproducts such as pyruvate, acetate, lactate etc. [18]. Therefore, phase-II and phase-III are the relevant phases for lysine synthesis. Previous studies have demonstrated that pentose phosphate pathway (PPP) and phosphoenolpyruvate carboxylase (PPC) shunt support substantial flux during lysine synthesis. Vallino and Stephanopoulos [10] have performed flux and nodal analysis to demonstrate that the branch point around the phosphoenolpyruvate (PEP) node is rigid, indicating a tight control of the lysine yield. This rigidity was due to the activation of PEP to OAA (oxaloacetate) reaction by AcCoA and inhibition of the same by aspartate. However, it should be noted that by eliminating rigidity around the PEP node, the rigidity might shift to other branch point such as glucose-6-phosphate (G6P) [10]. Thus, it is now clear that substantial alteration in flux partition at the three principle nodes namely PEP, pyruvate (PYR) and G6P in the metabolic pathway of the *C. glutamicum *is necessary to achieve high lysine yields. Although, flux analysis of *C. glutamicum *is well demonstrated for different fermentation stages, the effect of nitrogen and oxygen uptake rate on the productivity has not been quantified. We address this issue by analyzing the performance of the network at various oxygen and nitrogen uptake rates.

In the present work, we analyze the metabolic network of *C. glutamicum *using linear programming with the coefficients of the elementary modes as constraints. Further, we evaluate a feasible solution space for a given objective function of the network through a search in the space characterized by uptake rates of oxygen and nitrogen.

## Results

The metabolic network of *C. glutamicum *included the glucose phosphotransferase system, reactions of glycolytic pathway, reactions of TCA cycle and PPP as the core metabolism [see Additional file 1]. Also, carboxylation reaction for connection of PEP to OAA was included as it plays an important role for lysine synthesis. The ammonia assimilation was through the amino acid synthesis. Further, the oxidative phosphorylation accounted for NADH recycling with ATP synthesis, while the biomass formation was included as a stoichiometric reaction involving the internal metabolites [18]. It is reported that pyruvate carboxylase, pyruvate decarboxylase, malic enzyme and PEP decarboxykinase are also present in *C. glutamicum *[19]. *In vitro *studies indicate that the activities of the anaplerotic enzymes are negligible except that of PEP carboxylase, which is active in presence of glucose as the sole carbon source in the media [18,20-22]. Further, *in vivo *studies also indicate that these enzymes are inactive while growing on glucose alone [23]. These reactions are shown to be operational in the presence of lactate in the medium [19,20,24] due to pyruvate overflow in the system. Moreover, the glyoxylate shunt is active only in the presence of acetate in the media [25,26]. It is becoming clear that these important anaplerotic bioreactions are regulated in *C. glutamicum *in presence of lactate/acetate in the media. Since we analyze the system in presence of glucose alone, we do not include these anaplerotic reactions. Elementary modes represent the overall stoichiometry of the metabolic network in terms of inter conversion of external metabolites. This implies that the internal metabolites are at pseudo steady state levels and the accumulation rates of external metabolites can be used to evaluate the fluxes of the elementary modes (see method).

### Quantification of fluxes of elementary modes for *Corynebacterium glutamicum*

**Figure 1:**
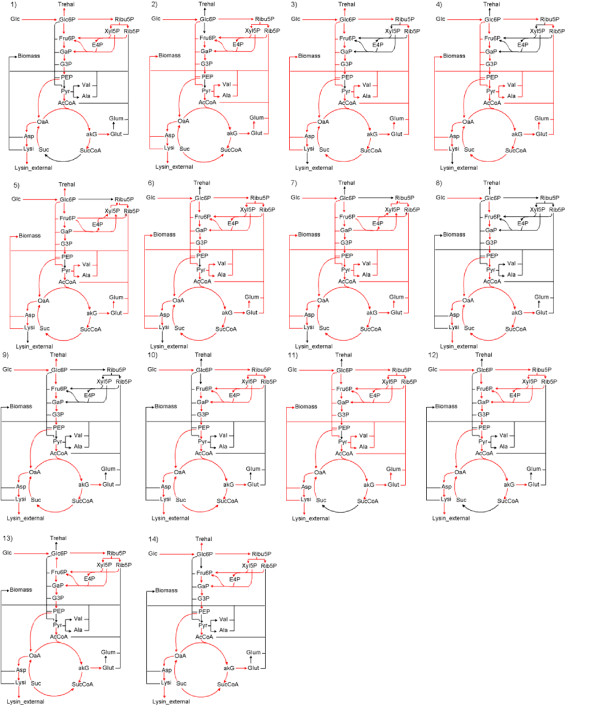
Graphical representation of the fourteen elementary modes obtained from the network of *Corynebacterium glutamicum*. Ammonia is consumed in glutamate synthesis reaction, while oxygen is consumed in the oxidative phosphorylation reaction (not shown in the figure). All elementary modes were associated with glucose, ammonia and oxygen as substrates. The detailed stoichiometries of these elementary modes are listed in Additional file 2.

Elementary modes were generated using the public domain python based software named *'ScrumPy'*, which was developed by M. G. Poolman & D. A. Fell (from Oxford Brooks University, UK). A total of 542 elementary modes were observed by incorporating all the possible extracellular metabolites of *C. glutamicum*. Experimental evidence suggested that the accumulation of lactate, acetate, pyruvate, alanine and valine were negligible during phase-I, II & III of lysine fermentation. These metabolites together accounted for less than 1.8 %, 0.87 % and 3.5 % of the glucose uptake rate at 11.5 h, 13.5 h and 15.8 h, respectively. Therefore, these metabolites were not considered as extracellular metabolites for the analysis. This modification resulted in only fourteen elementary modes [see Figure 1 and Additional file 2] by considering a total number of 38 metabolites [see Additional file 3] and 39 reactions [see Additional file 4]. Figure 1 shows the schematic representation of the reaction path for each of the fourteen elementary modes. All the elementary modes were associated with glucose, oxygen and ammonia as substrates, which essentially indicated an aerobic fermentation. The extracellular end products were biomass, lysine and trehalose. Biomass was synthesized using six elementary modes, while five elementary modes were associated with trehalose and nine with lysine production. However, lysine and biomass were simultaneously produced by two modes (mode '2' and '11'). Similarly, synthesis of lysine and trehalose was observed in three modes (mode '1', '9' and '14'), while synthesis of biomass and trehalose together were associated with two modes (mode '3' and '5'). Further, for the case of growth on glucose alone, a maximum theoretical yield of 63.5 moles per 100 moles of glucose was observed for lysine synthesis from the elementary mode '13' [Figure 1 and Additional file 2], while a maximum biomass yield of 124 moles per 100 moles of glucose was observed in mode '6'. These fourteen elementary modes were used to analyze the network at different time points during the fermentation. However, to evaluate the effect of other anaplerotic reactions on the maximum theoretical yield, elementary modes were also determined by including pyruvate carboxylase or malic enzyme instead of PEP carboxylase. The incorporation of this reaction resulted in a maximum theoretical lysine yield of 75 moles per 100 moles of glucose, which matches with the value reported in literature [27,28]. Moreover, incorporating all the anaplerotic reactions also yielded a maximum theoretical lysine yield of 75 mole %. The maximum yield of 63.5 mole % observed in our analysis was mainly due to the limitation of the PEP pool, which was channeled for the active transport of glucose and only a fraction of the PEP pool could be directed to OAA. This implies that lactate or acetate in combination with glucose in the media can enhance the lysine yield as all anaplerotic reactions are active [24,29].

Table 1 shows the accumulation rates of external metabolites at different time points during the course of fermentation as reported by Vallino [18]. Out of the twelve extracellular metabolites, only seven (glucose, oxygen, ammonia, biomass, lysine, trehalose and carbon dioxide) were used for the analysis as the accumulation rates of remaining external metabolites (acetate, alanine, lactate, pyruvate and valine) were negligible. At this point, it was relevant to question the minimum requirement of the accumulation rates of the external metabolites for analysis of the network for proper closure of mass balance. This issue was examined by linear programming using the coefficient matrix of the fourteen elementary modes as constraints during the course of fermentation (see method section). The uptake rates of external metabolites (glucose, oxygen and ammonia) and the synthesis rate of lysine were decision variables to the linear programming solver with maximization of biomass as an objective function. Table 2 shows the comparison of accumulation rates obtained through elementary mode analysis with experimental observations. The predicted values of biomass, trehalose and carbon dioxide matched exactly with the experimental values. Analysis using more than four accumulation rates as decision variables also predicted the same result for the extracellular accumulation rates. However, using only three accumulation rates as decision variables (i.e. glucose, oxygen and ammonia), the analysis resulted in a different solution depending on the choice of objective function, which did not match the experimental values. Thus, four measurements were essential to uniquely quantify the remaining accumulation rates of the extracellular metabolites using elementary modes.

**Table 1:** Normalized and absolute (within bracket) extracellular metabolites accumulation rates

**Extracellular metabolites**	**Accumulation rates (mM/h)**		
	**11.5 h**	**13.5 h**	**15.8 h**
Ac	0.16 (0.04)	0.028 (0.01)	0.35 (0.1)
Ala	0.68 (0.16)	0.056 (0.02)	0.14 (0.04)
Biomass	90.43 (22.7)	50.56 (17.9)	23.5 (6.7)
CO_2_	251.7 (63.2)	218.6 (77.4)	257.9 (73.5)
Glc	-100.0 (-25.1)	-100.0 (-35.4)	-100.0 (-28.5)
Lac	0.24 (0.06)	0.056 (0.02)	0.53 (0.15)
Lysi	0.16 (0.04)	30.23 (10.7)	29.37 (8.37)
NH_3_	-67.7 (-17.0)	-96.9 (-34.3)	-78.25 (-22.3)
O_2_	-230.67 (-57.9)	-177.4 (62.8)	-221.4 (-63.1)
Pyr	0.52 (0.13)	0.169 (0.06)	0.46 (0.13)
Trehal	2.51 (0.63)	2.54 (0.9)	5.96 (1.7)
Val	0.24 (0.06)	0.565 (0.2)	2.1 (0.6)

The data was taken from Vallino [18]. It can be noted that during 11.5 – 15.8 h, the accumulation rates of acetate, alanine, lactate, pyruvate, valine were negligible. Thus, only the uptake rates of glucose, ammonia and oxygen and accumulation rates of biomass, lysine, trehalose and carbon dioxide were considered for the analysis. Negative sign indicates the uptake rate of external metabolite.

**Table 2:** Comparison of experimental data and predicted values of accumulation rates of various metabolites at different time points

**Accumulation rate (mM/h)**	**Biomass**		**Trehalose**		**CO_2_**	
Time (h)	Expt.	Predicted	Expt	Predicted	Expt	Predicted
11.5	90.43	91.55	2.51	2.7	251.79	249.0
13.5	50.53	49.51	2.54	2.45	218.6	217.46
15.8	23.5	26.5	5.96	6.32	257.89	256.0

Glucose, ammonia, oxygen and lysine were the decision variables to the linear programming optimizer with the objective function of maximization of biomass; Values predicted were for the accumulation rates of biomass, trehalose and carbon dioxide. It was observed that the choice of decision variables of accumulation rates and objective function yielded the same prediction of extracellular accumulation rates.

To study the effect of the maximization criteria, the network was analyzed using other objective functions. The accumulation rates of glucose, lysine, trehalose and biomass were used with maximization of carbon dioxide as the objective function. Table 3 shows a comparison of the accumulation rates obtained through the elementary mode analysis and the experimental values for accumulation of ammonia, oxygen and carbon dioxide. It is clear from Table 2, 3 that the solution matched with the experimental data. The analysis indicated that the form of the objective function did not affect the prediction for the accumulation rates of extracellular metabolites if a minimum of four accumulation rates were considered for the analysis. However, it was noted from the above analysis that the predictions of the accumulation rates were accurate using a minimum of four accumulation rates, but the fluxes of the elementary modes were not unique and were dependent on the choice of the objective function. For a set of decision variables including four extracellular accumulation rates, there could be multiple flux maps of elementary modes. To verify the above, different feasible flux maps of the elementary modes were obtained by invoking seven objective functions (including glucose, oxygen, ammonia, carbon dioxide, lysine, trehalose and biomass) with both maximization and minimization criteria. It should be noted that in all the cases the extracellular accumulation rates yielded were same as shown in Table 2, 3, indicating a perfect match with the experimental data. However, the analysis yielded ranges of allowable flux values for an individual elementary mode. Based on the criteria of the objective function, a set of flux values for the elementary modes (in the allowable flux range) would match the experimental data. Moreover, each of the feasible set of flux values of the fourteen elementary modes yielded identical flux distribution of the original network (results not shown). Thus, the four measurements were sufficient to uniquely characterize the internal fluxes of the original network but yielded different feasible sets of fluxes for the elementary modes depending upon the objective function. Further, the internal flux distribution of the original network matched with that obtained through FBA (result not shown).

**Table 3:** Comparison of experimental data and predicted values of accumulation rates of various metabolites at different time points

**Accumulation rate (mM/h)**	**NH_3_**		**O_2_**		**CO_2_**	
Time (h)	Expt.	Predicted	Expt	Predicted	Expt	Predicted
11.5	-67.72	-66.88	-230.68	-237.1	251.79	255.2
13.5	-96.89	-97.67	-177.4	-172.48	218.6	212.74
15.8	-78.24	-76.02	-221.4	-236.77	257.89	270.8

Glucose, lysine, trehalose and Biomass were the decision variables to the linear programming optimizer with the objective function of maximization of carbon dioxide. Values predicted were for the uptake rates of ammonia, oxygen and accumulation rate of carbon dioxide. It was observed that the choice of decision variables of accumulation rates and objective function yielded the same prediction of extracellular accumulation rates.

**Figure 2:**
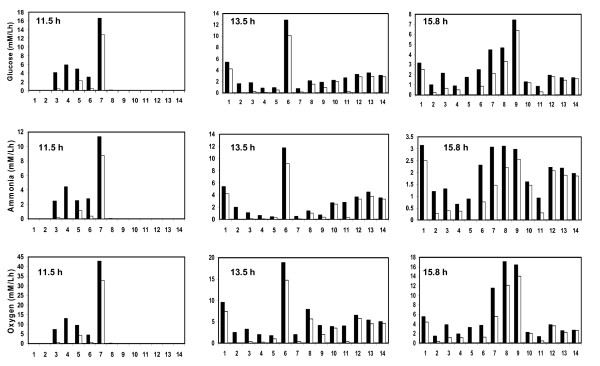
Histogram of feasible range of flux values for substrate consumption rates for the metabolic network of *C. glutamicum *through individual elementary mode. The figure depicts the ranges of absolute values for uptake values of glucose, ammonia and oxygen. The fluxes were estimated by linear programming using various objective functions as described in the text. Uptake rates of glucose, ammonia, oxygen and accumulation rates of lysine were the decision variables to the optimizer. The fluxes were estimated at different time points (11.5 h, 13.5 h and 15.8 h) during the course of the fermentation. Black box indicates the maximum feasible value; white box indicates the minimum feasible value. The number on the x-axis indicates the serial number of the elementary modes as indicated in Figure 1 or in Additional file 2.

The ranges of the allowable flux values for an individual elementary modes was used to determine its contribution towards uptake rates of substrates (glucose, ammonia, oxygen) and formation of products (biomass, lysine and trehalose). Figure 2 shows the contribution of the 14 elementary modes towards uptake rates of glucose, ammonia and oxygen at 11.5 h, 13.5 h and 15.8 h. It can be noted that the contribution of an elementary mode towards an extracellular metabolite was represented as a range of feasible set. For example, at 11.5 h, it can be seen that mode '4' was used to consume glucose in the range of 0 to 6 mM/Lh, while mode '7' had a range of 13 to 16.5 mM/Lh. Although the elementary modes have a set of feasible flux values, the maximum and minimum values show a distinct trend for a given time point. Therefore, we discuss the result after taking an average of the maximum and minimum feasible values for each of the elementary mode. At t = 11.5 h, 77 % of the glucose uptake rate was associated towards formation of biomass (through modes '4', '6' and '7'; see Figure 2). Further, most of the glucose (about 58 %) was consumed through mode '7' (this mode is operational through TCA cycle). The remaining 23% of the net glucose uptake rate resulted in biomass and trehalose formation (through modes '3' and '5'). All other modes were effectively inactive. This indicated that at t = 11.5 h, the carbon and nitrogen in the medium was effectively used towards biomass formation. At t = 13.5 h, it was observed that all the modes were operational and 35 % of the total glucose uptake rate contributed to biomass synthesis alone (through modes '4', '6' and '7'). Further, about 26 % of the glucose uptake rate contributed towards lysine and trehalose synthesis (mode '1', '9' and '14') and another 30 % towards the synthesis of lysine alone (mode '8', '10', '12' and '13'). It should be noted that mode '7' dominated at t = 11.5 h, while mode '6' (operational through pentose phosphate pathway) dominated at t = 13.5 h. At t = 15.8 h, the glucose uptake rate towards biomass decreased to 18 %, while increased towards lysine and trehalose to 70 %. The flux distribution of elementary modes related to ammonia and oxygen uptake rate for different time points were similar to the distribution of glucose since all the elementary modes were associated with glucose, oxygen and ammonia as substrates (Figure 2).

**Figure 3:**
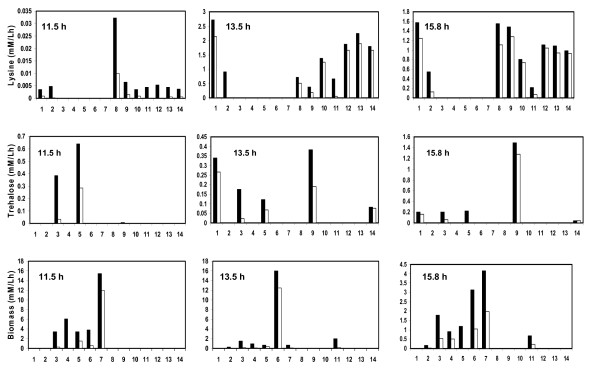
Histogram of feasible range of flux values for the product formation rates for the metabolic network of *C. glutamicum *through individual elementary mode. The figure depicts the ranges of absolute values for formation of biomass, lysine and trehalose. The details are the same as given in the legend of Figure 2.

Figure 3 shows the fluxes through the fourteen modes that contributed towards biomass, lysine and trehalose formation. At t = 11.5 h, 59 % of biomass formation was through the glycolysis and TCA cycle (through mode '7'), while remaining 41 % of biomass formation was almost equally distributed among the modes '3', '4', '5' and '6'. It was noted that 19 % of total biomass formation also resulted with trehalose formation. The glucose uptake rate, at t = 13.5 h through mode '6', contributed towards most of the biomass synthesis. Further, at t = 15.8 h, biomass synthesis reduced to 26 % as compared to that observed at t = 11.5 h. Elementary modes associated with only biomass formation contributed to 85 % at 13.5 h and 72% at 15.8 h of the total biomass synthesis rate. Further, all elementary modes (mode '8', '10', '12', '13') associated with lysine synthesis were inactive at 11.5 h. Only lysine producing modes contributed to about 52 % of lysine production at later stage of fermentation, while 50 % of the lysine production rate was associated with modes having lysine and trehalose. The synthesis of trehalose was accompanied with biomass synthesis modes at t = 11.5 h, whereas, trehalose synthesis was accompanied by lysine synthesis at later times. Thus, the analysis indicated that during the fermentation different sets of elementary modes were operational at different time points.

### Simulation of feasible phenotypic space

As discussed above, the fluxes through individual modes were evaluated using the experimentally determined accumulation rates of glucose, ammonia, oxygen and lysine. However, the network can also be simulated by considering only the three uptake rates of substrates (i.e. glucose, ammonia and oxygen) as decision variables with a maximization criterion (maximization of biomass or that of lysine) to study the metabolic capability of the organism at various nutrient uptake rates. Thus, the network of *C. glutamicum *was simulated with the normalized glucose uptake rate equal to 100 moles/Lh with various combinations of uptake rates of ammonia and oxygen.

**Figure 4:**
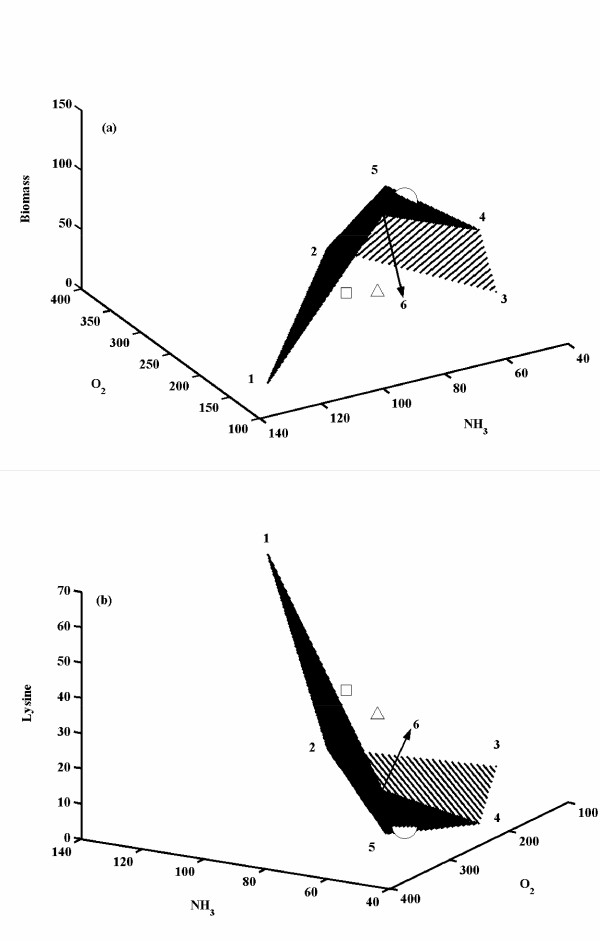
Optimized solution spaces for biomass and lysine synthesis rates in *C. glutamicum *for varying ammonia and oxygen uptake rates. The uptake rate of glucose was fixed at 100 moles/Lh. The choice of the objective function was maximization of biomass. **(a) **shows the biomass synthesis rates at various ammonia and oxygen consumption rates. The numbered points represent, **1**: (155, 127, 0); **2**: (365, 67, 0); **3**: (220, 40, 0); **4**: (192, 51, 69); **5**: (256, 69, 94); **6**: (146, 91, 124) in the order given by oxygen and ammonia uptake rates and biomass formation rate, respectively. **(b) **shows the lysine synthesis rates at various ammonia and oxygen consumption rates. The numbered points indicate, **1**: (155, 127, 63.5); **2**: (365, 67, 33.5); **3**: (220, 40, 20); **4**: (192, 51, 0); **5**: (256, 69, 0); **6**: (146, 91, 0) in the order given by oxygen uptake rate, ammonia uptake rate and lysine formation rate, respectively. The experimental values were reported by Vallino [18] at 11.5 h, 13.5 h and 15.8 h and were plotted onto the graph. '○' indicates experimental data at 11.5 h; '□' indicates experimental data at 13.5 h and '△'indicates experimental data at 15.8 h.

Figure 4 shows the optimized solution space for synthesis rates of biomass and lysine with varying normalized uptake rates of oxygen and ammonia for the criteria of maximization of biomass. The feasibility of oxygen uptake rate was found to be in the range of 146 – 365. Also, feasible ammonia uptake rate was found to be in the range of 40 – 127 relative to glucose uptake rate. The maximum biomass formation rate was 123.64 for a normalized oxygen and ammonia uptake rate of 146 and 91, respectively with an equivalent lysine production rate being zero (Figure 4a). However, at a normalized oxygen and ammonia uptake rate of 155 and 127, respectively, a maximum lysine of 63.5 was observed with biomass synthesis being zero (Figure 4b). It was interesting to note that the solution space had an unique maximum (i.e. with 124 flux towards biomass), while it showed several minima (with biomass formation rate = 0). The analysis also resulted in unique maximum value for the lysine synthesis rate (Figure 4b). The experimental observation of biomass and lysine formation rates at 11.5 h, 13.5 h, and 15.8 h were also plotted onto this solution space. It can be seen that at t = 11.5 h, the biomass formation rate lay on the edge of the solution space indicating that the cells were maximizing biomass at this log phase of fermentation, whereas at t = 13.5 h and 15.8 h the biomass synthesis rates did not lie on the solution space. In the equivalent lysine synthesis space (for the same criteria of biomass maximization), the experimental point observed at t = 11.5 h was at the bottom indicating low rate of lysine synthesis, whereas the other two points were not inside the solution space as observed for the biomass. It has been reported that lysine synthesis was inhibited by the threonine concentration in the media. The time points at which the switch from maximum biomass synthesis to a suboptimal biomass synthesis occurs that can be related to the depletion of threonine concentration in the media as observed by Vallino [18]. In summary, this analysis indicated that the maximization of biomass was valid only in the early exponential growth phase.

**Figure 5:**
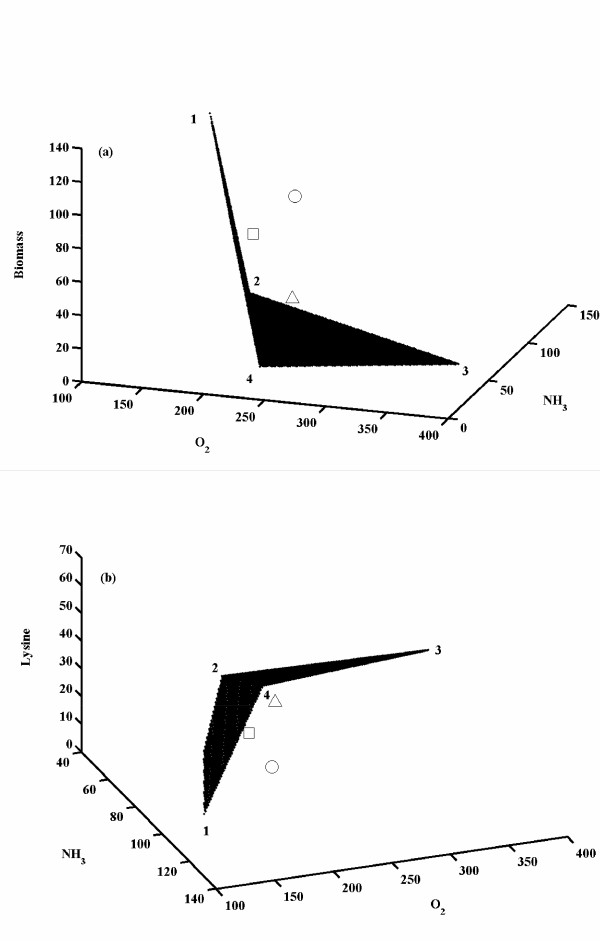
Optimized solution spaces for biomass and lysine synthesis rates in *C. glutamicum *for varying ammonia and oxygen uptake rates. The uptake rate of glucose was fixed at 100 moles/Lh. The choice of the objective function was maximization of lysine. **a **shows the biomass synthesis rates at various ammonia and oxygen consumption rates. The numbered points represent, **1**: (146, 91, 124) **2**: (155, 127, 0); **3**: (365, 67, 0); **4**: (220, 40, 0); in the order given by oxygen and ammonia uptake rates and biomass formation rate, respectively. **b **shows the lysine synthesis rates at various ammonia and oxygen consumption rates. The numbered points indicate, **1**: (146, 91, 0) **2**: (155, 127, 63.5); **3**: (365, 67, 33.5); **4**: (220, 40, 20); in the order given by oxygen and ammonia uptake rates and biomass formation rate respectively. '○' indicates experimental data at 11.5 h; '□' indicates experimental data at 13.5 h and '△'indicates experimental data at 15.8 h.

Figure 5 shows the fluxes towards biomass and lysine synthesis for various relative oxygen and ammonia uptake rates (relative to glucose uptake rate of 100) under the criteria of maximization of lysine. The solution space was found to be distinct as compared to the criteria of maximization of biomass, while the feasible set of ammonia and oxygen uptake rates were same as in the case of maximization criteria of biomass. It was also observed that none of the experimental points lay on the solution space obtained through maximization of lysine. This clearly indicates that the metabolic regulations that have evolved in *C. glutamicum *do not tend to maximize lysine synthesis. However, it was observed that on the edges of the surface of the three dimensional solution space, the form of the objective function did not matter indicating that solution obtained was independent of the criteria of maximization. In such a condition, the reaction from PEP to PYR was not operational and carbon for glycolysis and PPP was channeled through PEP carboxylase to OAA. This implies that TCA cycle and lysine synthesis reactions were operational through PEP carboxylase at points lying on the edges of the solution space. The carboxylase enzyme, which was present in five elementary modes (mode '4', '7', '8', '10', '12'), would not be operational on the edges of the solution space. However, for a non-edge point in the solution space, the maximization criteria yielded different set of fluxes. For example, at glucose, ammonia and oxygen uptake rates of 100, 67 and 332, respectively with the criteria of maximization of biomass yielded a biomass formation rate of 28.6 and lysine formation rate of 23. In this case, the flux from PEP to PYR was highly active (around 60) with no flux through pentose phosphate pathway, while with the criteria of maximization of lysine, biomass formation rate was zero and lysine synthesis rate was higher at 33.5. In this non-edge solution, flux from PEP to PYR and PPP were operational to maximize lysine synthesis, which was required for NADPH balancing.

## Discussion

We have quantified the metabolic network of *C. glutamicum *using elementary modes by linear optimization. The analysis was used to determine the feasible set of flux values for the elementary modes. The individual stoichiometric combination of various substrates towards specific product can be evaluated using this methodology. The linear optimization was used to determine the fluxes of elementary modes using accumulation rates obtained through experiments. In case of *C. glutamicum*, only four measurement values were essential for the closure of molar balance. In such a situation, the solution for predicting the accumulation rates of remaining external metabolites was independent of the objective function. This, however, did not ensure a unique flux distribution for the various elementary modes and provided a set of feasible ranges. By utilizing lesser number of accumulation rates in the linear optimization strategy, one could determine the capability of the network towards achieving a specific objective function. We used the uptake rates of three substrates (i.e. glucose, ammonia and oxygen) to evaluate the capability of the network to produce maximum biomass or lysine. The solution space bounds the accumulation rate of a metabolite for a specific substrate combination. The stoichiometry of the elementary modes also enforces a constraint on the uptake rates of the substrates. For example, in *C. glutamicum *for a given glucose (100) and ammonia (85) uptake rates, the oxygen uptake rate was bounded within a range of 153 – 302. This also reflects the constraint of the aerobic and anaerobic routes that may be used for balancing the oxidative potential (i.e. balancing of NADH/NAD^+^). Similarly, the demand for balancing of NADPH/NADP^+ ^may switch on the elementary modes containing the pentose phosphate pathway. In *C. glutamicum*, this was observed in the modes involving lysine synthesis. Also, for a given glucose and oxygen uptake rate, the ammonia uptake gets bounded, resulting in a constraint on the productivity of lysine. The feasible solution space was equivalent to the phenotypic phase plane as reported by Edwards et al. [30], where a phenotypic phase plane was evaluated for the growth of *E. coli *on glucose and acetate at various oxygenation levels. Here, we have similarly attempted to obtain optimal growth and lysine synthesis rates at various oxygenation levels and ammonia uptake rates.

The methodology presented here is useful in deciphering the capability of an organism for a given substrate combination. For example, the maximum yield coefficient (ratio of accumulation rate of a product and that of biomass) can be evaluated for a specific nutrient combination. Further, the analysis can yield the operational elementary modes at different points of the feasible solution space. In case of *C. glutamicum*, the biomass formation rate was highest (124) at ammonia and oxygen uptake rates of 91 and 146, respectively. In this case, elementary modes numbered as '5', '6', '7', were mainly functional and operated through the routes using glycolysis and TCA cycle with carbon dioxide as only the byproduct. Interestingly, we obtained the same result with the objective function of maximization of lysine. This implies that a minimum oxygen uptake of 146 is essential during the metabolism of *C. glutamicum*. A similar behavior in the flux distribution of each elementary mode was observed at the extreme points of the feasible set of oxygen and ammonia consumption rate. It was noted that for a maximum feasible ammonia uptake rate (127), the lysine synthesis rate was at a maximum with the elementary mode '13' being functional. This elementary mode included reactions involving PPP, PPC, TCA cycle and lysine synthesis.

Evaluation of the fluxes of elementary modes can therefore provide functional insights about any metabolic network. The operational limitation caused by ATP and NADH balancing was inherently captured using the elementary modes. The stoichiometric distribution towards a targeted product using a specific elementary mode provides insights into the route that needs engineering to enhance productivity. Further, the flux distribution through the elementary modes can yield overall capability of the organism in terms of growth, product formation and substrate uptake rates for a given media composition.

## Conclusion

We have demonstrated the use of elementary modes with the help of linear optimization in quantifying metabolic networks. This methodology was used to quantify the network of *C. glutamicum *for lysine synthesis. Analysis in conjunction with experimental accumulation rates gives insight into the contribution of various elementary modes towards the accumulation rates of extracellular metabolites. In *C. glutamicum*, the elementary modes associated with biomass formation were operational at the initial experimental growth phase, while modes associated with lysine synthesis switch on at later phase of fermentation. The methodology was also used to determine the feasible solution space for a given set of substrate uptake rates. Such an approach is generic in nature and can be used to determine the optimality of the accumulation rates of a metabolite in any given system.

## Methods

Elementary modes for the network were obtained using *'ScrumPy' *software [31]. The input file of the reaction set to the software is provided as a .spy file [see Additional file 4]. The accumulation rates of extracellular metabolites can be represented through the fluxes in the elementary modes with equivalent stoichiometry of the elementary modes. The matrix representation of the same can be given as

*S*·*E *= *M *    (1)

where, *M *is vector representing the accumulation rates of the external metabolites and *S *is the matrix, indicating the stoichiometry of the elementary modes. The elements of *E*{*e*_1_, *e*_2_, *e*_3_,.........*e*_*n*_} represents the vector of unknown fluxes of the elementary modes. The elements of vector *E *can be evaluated for a given set of measurements of accumulation rates (elements of *M*). Due to the paucity of measurements, a linear optimization technique can be employed to converge onto a feasible solution. Mathematically, the linear optimization formulation can be represented as:

Max(*M*_*i*_)

such that,

S*E = *M *^•^

0 ≤ *e*_*i*_≤ ∞     for all the elements     (2)

*M*_*i *_is the accumulation rate of a specific metabolites (such as biomass or carbon dioxide). Also, S* is stoichiometric matrix and M* is know vector of the accumulation rates of extracellular metabolites, wherein the rows of the *i*^*th *^external metabolite are eliminated from *S *and *M*, respectively. It should be noted that the above approach of using linear programming to evaluate fluxes is similar to the methodology used in FBA. FBA uses the stoichiometry of the original network for the analysis, while we use the stoichiometries of the elementary modes. The methodology has been explained using an illustrative example [see Additional file 5]

## Authors' contributions

KG and KVV conceived the methodology and designed the experiments. KG performed the various *in silico *experiments. KG & KVV conceptualized the manuscript. All authors have read and approved the final manuscript.

## Supplementary Material

Additional File 1 Reactions involved in the metabolic network of *C. glutamicum*. Lists all the reactions in the metabolic network used for the analysis. Click here for file

Additional File 2 Elementary modes obtained for the network of *C. glutamicum*. Lists the elementary modes obtained using "ScrumPy" software. Click here for file

Additional File 3 Abbreviations used for various metabolites. Lists the abbreviations used for the metabolites present in the network. Click here for file

Additional File 4 Input file used to generate the elementary modes. The input .spy file used in "ScrumPy" software to obtain elementary modes Click here for file

Additional File 5 Demonstration of the methodology using a simple illustrative example. The optimization methodology is applied to an illustrative example to demonstrate the steps used for obtaining the solution. Click here for file
