# Supplementary material for: Analysis of optimal phenotypic space using elementary modes as applied to Corynebacterium glutamicum
Source: BMC Bioinformatics. 2006 Oct 12;7:445. doi: 10.1186/1471-2105-7-445 (PMC1617123; doi:10.1186/1471-2105-7-445)
Supplement: Additional File 1 — Reactions involved in the metabolic network of C. glutamicum. Lists all the reactions in the metabolic network used for the analysis. [file 1471-2105-7-445-S1.doc]

### Additional file 1 – Reactions involved in the metabolic network of *C. glutamicum*

The chemical equations are representing the metabolic network of *C. glutamicum* as follows; where ‘$\leftrightarrow$’ represents the reversible reaction and ‘’ represents the irreversible reaction. “X” superscript denotes the external metabolites [18]. Stiochiometries of the chemical equations are in terms of moles.

**Glucose Phosphotransferase System**

1. X_GLC + PEP GLC6P + PYR

**Storage Compounds; Trehalose**

2. 2 GLC6P + ATP $\leftrightarrow$ TREHAL + ADP

**EMP Pathway**

3. GLC6P $\leftrightarrow$FRU6P

4. FRU6P + ATP 2 GAP + ADP

5. GAP + ADP + NAD $\leftrightarrow$ NADH + G3P + ATP

6. G3P $\leftrightarrow$ PEP + H2O

7. PEP + ADP ATP + PYR

8. PYR + NADH $\leftrightarrow$ LAC + NAD

**Carboxylation reaction**

9. PEP + CO2 OAA

**TCA Cycle**

10. PYR + COA + NAD ACCOA + CO2 + NADH

11. ACCOA + OAA + H2O + NADP$\leftrightarrow$ AKG + COA + NADPH + CO2

12. AKG + COA + NAD SUCCOA + CO2 + NADH

13. SUCCOA + ADP $\leftrightarrow$ SUC + COA + ATP

14. SUC + H2O + FAD +NAD $\leftrightarrow$ FADH + OAA + NADH

**Acetate Production or Consumption**

15. ACCOA + ADP $\leftrightarrow$ AC + COA + ATP

**Glutamate, Glutamine, Alanine, and Valine Production**

16. NH3 + AKG + NADPH $\leftrightarrow$ GLUT + H2O + NADP

17. GLUT + NH3 + ATP GLUM + ADP

18. PYR + GLUT ALA + AKG

19. 2 PYR + NADPH + GLUT VAL + CO2 + H2O + NADP + AKG

**Pentose Phosphate Pathway**

20. GLC6P + H2O + 2 NADP RIBU5P + CO2 + 2 NADPH

21. RIBU5P $\leftrightarrow$ RIB5P

22. RIBU5P $\leftrightarrow$ XYL5P

23. XYL5P + RIB5P $\leftrightarrow$ SED7P + GAP

24. SED7P + GAP $\leftrightarrow$FRU6P + E4P

25. XYL5P + E4P $\leftrightarrow$ FRU6P + GAP

**Oxidative Phosphorylation**

26. 2 NADH + O2 + 4 ADP 2 H2O + 4 ATP + 2 NAD

27. 2 FADH + O2 + 2 ADP 2 H2O + 2 ATP + 2 FAD

**Asparate Amino Acid Family**

28. OAA + GLUT $\leftrightarrow$ ASP + AKG

29. ASP + PYR + 2 NADPH + ATP AKP + 2 NADP + ADP + H2O

30. AKP + SUCCOA + H2O + GLUT MDAP + COA + AKG + SUC

31. MDAP LYSI + CO2

**ATP Dissipation**

32. ATP ADP

**Biomass Synthesis**

33. 30 PYR + 21 GLC6P + 7 FRU6P + 150 G3P + 52 PEP + 13 GAP + 332 ACCOA + 126 RIB5P + 80 ASP + 33 LYSI + 446 GLUT + 25 GLUM + 54 ALA + 40 VAL + 100 NADPH + 3000 ATP 1000 BIOMAS + 143 CO2 + 100 NADP + 332 COA + 364 AKG + 3000 ADP

**External metabolites**

34. X_O2 O2

35. X_NH3 NH3

36. BIOMASS X_BIOMASS

37. LYSI X_LYSI

38. TREHAL X_TREHAL

39. CO2 X_CO2
