# Supplementary material for: Analysis of optimal phenotypic space using elementary modes as applied to Corynebacterium glutamicum
Source: BMC Bioinformatics. 2006 Oct 12;7:445. doi: 10.1186/1471-2105-7-445 (PMC1617123; doi:10.1186/1471-2105-7-445)
Supplement: Additional File 2 — Elementary modes obtained for the network of C. glutamicum. Lists the elementary modes obtained using "ScrumPy" software. [file 1471-2105-7-445-S2.doc]

### Additional file 2: Elementary modes obtained for the network of *C. glutamicum*

Elementary modes for the network of *C. glutamicum*, where serial number identifies a specific elementary mode used in the text. For abbreviation see Additional file 3.

| **Sl. No** | **Reaction Stoichiometry of the elementary modes** |
| --- | --- |
| 1 | 192 GLC + 336 O2 + 192 NH3 12 TREHAL + 96 LYSI + 816 H2O + 432 CO2 |
| 2 | 11892 GLC + 18237 O2 + 14552 NH3 6540 LYSI + 49808 H2O + 2000 BIOMAS + 25174 CO2 |
| 3 | 912216 GLC + 1637634 O2 + 562304 NH3 86141 TREHAL + 3938420 H2O + 764000 BIOMAS + 1789288 CO2 |
| 4 | 369967 GLC + 818817 O2 + 281152 NH3 1969210 H2O + 382000 BIOMAS + 894644 CO2 |
| 5 | 28920 GLC + 55470 O2 + 14720 NH3 3725 TREHAL + 125780 H2O + 20000 BIOMAS + 59440 CO2 |
| 6 | 1186920 GLC + 1730295 O2 + 1081920 NH3 5020680 H2O + 1470000 BIOMAS + 2022090 CO2 |
| 7 | 10735 GLC + 27735 O2 + 7360 NH3 62890 H2O + 10000 BIOMAS + 29720 CO2 |
| 8 | 75 GLC + 275 O2 + 50 NH3 25 LYSI + 550 H2O + 300 CO2 |
| 9 | 20 GLC + 44 O2 + 8 NH3 4 TREHAL + 4 LYSI + 88 H2O + 48 CO2 |
| 10 | 18 GLC + 31 O2 + 22 NH3 11 LYSI + 80 H2O + 42 CO2 |
| 11 | 21040 GLC + 31384 O2 + 22112 NH3 5168 LYSI + 88608 H2O + 16000 BIOMAS + 39728 CO2 |
| 12 | 56 GLC + 112 O2 + 64 NH3 32 LYSI + 272 H2O + 144 CO2 |
| 13 | 44 GLC + 68 O2 + 56 NH3 28 LYSI + 184 H2O + 96 CO2 |
| 14 | 38 GLC + 62 O2 + 44 NH3 TREHAL + 22 LYSI + 160 H2O + 84 CO2 |
