# Supplementary material for: Analysis of optimal phenotypic space using elementary modes as applied to Corynebacterium glutamicum
Source: BMC Bioinformatics. 2006 Oct 12;7:445. doi: 10.1186/1471-2105-7-445 (PMC1617123; doi:10.1186/1471-2105-7-445)
Supplement: Additional File 3 — Abbreviations used for various metabolites. Lists the abbreviations used for the metabolites present in the network. [file 1471-2105-7-445-S3.doc]

**Additional file 3 - Abbreviations used for various metabolites**

| ADP | Adenosine diphosphate |
| --- | --- |
| AKG | $\alpha$-Ketoglutaric acid |
| AKP | 2-Amino-6-ketopimelate |
| ALA | Alanine |
| ASP | Aspirate |
| ATP | Adenosine triphosphate |
| BIOMASS | Biomass |
| CO2 | Carbon dioxide |
| E4P | Erythrose­-4-phosphate |
| FAD | Flavin adenine dinucleotide (oxidized) |
| FADH | Flavin adenine dinucleotide (reduced) |
| FRU6P | Fructose-6-phosphate |
| GAP | Glyceraldehydes-3-phosphate |
| GLC | Glucose |
| GLC6P, GL6P | Glucose-6-phosphate |
| GLUM | Glutamine |
| GLUT | Glutamate |
| G3P | 3-Phosphoglycerate |
| H2O | Water |
| ISOCIT | Isocitrate |
| LYSI | Lysine |
| MAL | Malate |
| MDAP | *Meso*-Diaminopimelate |
| NAD | Nicotinamide adenine dinucleotide (oxidized) |
| NADH | Nicotinamide adenine dinucleotide (reduced) |
| NADP | Nicotinamide adenine dinucleotide phosphate (oxidized) |
| NADPH | Nicotinamide adenine dinucleotide phosphate (reduced) |
| NH3 | Ammonia |
| OAA | Oxalate |
| PEP | Phosphoenolpyruvate |
| PPP | Pentose phosphate pathway |
| PPC | PEP carboxylase |
| PYR | Pyruvate |
| RIB5P | Ribose-5-phosphate |
| RIBU5P | Ribulose-5-phosphate |
| SUC | Succinate |
| SUCCOA | Succinyl coenzyme A |
| TCA | Tricarbxylic Acid |
| TREHAL | Trehalose |
| VAL | Valine |
| XYL5P | Xylulose-5-phosphate |
