# Supplementary material for: Analysis of optimal phenotypic space using elementary modes as applied to Corynebacterium glutamicum
Source: BMC Bioinformatics. 2006 Oct 12;7:445. doi: 10.1186/1471-2105-7-445 (PMC1617123; doi:10.1186/1471-2105-7-445)
Supplement: Additional File 4 — Input file used to generate the elementary modes. The input .spy file used in "ScrumPy" software to obtain elementary modes [file 1471-2105-7-445-S4.doc]

**Additional file 4 – Input file used to generate the elementary modes**

Format of the file has been described by Poolman et al. [17].

Ammoniauptake:

X_NH3 -> NH3

~

Oxygenuptake:

X_O2 -> O2

~

GlucosePhosphotransferase:

X_GLC + PEP -> GLC6P + PYR

~

Glucosestorage:

2 GLC6P + ATP <> TREHAL + ADP

~

EMPPathway:

GLC6P <> FRU6P

~

E1:

FRU6P + ATP -> 2 GAP + ADP

~

E2:

GAP + ADP + NAD <> NADH + G3P + ATP

~

E3:

G3P <> PEP + H2O

~

E4:

PEP + ADP -> ATP + PYR

~

E5:

PYR + NADH <> LAC + NAD

~

Carboxilationreaction:

PEP + CO2 -> OAA

~

TCACycle:

PYR + COA + NAD -> ACCOA + CO2 + NADH

~

TC7:

ACCOA + OAA + H2O + NADP <> COA + AKG + NADPH + CO2

~

TC3:

AKG + COA + NAD -> SUCCOA + CO2 + NADH

~

TC4:

SUCCOA + ADP <> SUC + COA + ATP

~

TC8:

SUC + H2O + FAD + NAD <> FADH + OAA + NADH

~

Acetateproduction:

ACCOA + ADP <> AC + COA + ATP

~

Glutamineproduction:

NH3 + AKG + NADPH <> GLUT + H2O + NADP

~

GLU1:

GLUT + NH3 + ATP -> GLUM + ADP

~

Alaninesyntesis:

PYR + GLUT -> ALA + AKG

~

Valinesynthesis:

2 PYR + NADPH + GLUT -> VAL + CO2 + H2O + NADP + AKG

~

PentosePhosphatepathway:

GLC6P + H2O + 2 NADP -> RIBU5P + CO2 + 2 NADPH

~

PP7:

RIBU5P <> RIB5P

~

PP8:

RIBU5P <> XYL5P

~

PP3:

XYL5P + RIB5P <> SED7P + GAP

~

PP4:

SED7P + GAP <> FRU6P + E4P

~

PP5:

XYL5P + E4P <> FRU6P + GAP

~

Oxidativephosphorylation:

2 NADH + O2 + 4 ADP -> 2 H2O + 4 ATP + 2 NAD

~

OXI1:

2 FADH + O2 + 2 ADP -> 2 H2O + 2 ATP + 2 FAD

~

Asparateacidfamily:

OAA + GLUT <> ASP + AKG

~

AS1:

ASP + PYR + 2 NADPH + ATP -> AKP + 2 NADP + ADP + H2O

~

AS2:

AKP + SUCCOA + H2O + GLUT -> MDAP + COA + AKG + SUC

~

AS3:

MDAP -> LYSI + CO2

~

Biomassynthesis:

30 PYR + 21 GLC6P + 7 FRU6P + 150 G3P + 52 PEP + 13 GAP + 332 ACCOA + 126 RIB5P + 80 ASP + 33 LYSI + 446 GLUT + 25 GLUM + 54 ALA + 40 VAL + 100 NADPH + 3000 ATP -> 1000 BIOMAS + 143 CO2 + 100 NADP + 332 COA + 364 AKG + 3000 ADP

~

ATPDissipation:

ATP -> ADP

~

Biomassexternal:

BIOMAS -> X_BIOMAS

~

CO2external:

CO2 -> X_CO2

~

Lysintransport:

LYSI -> X_LYSI

~

Trehalexternal:

TREHAL -> X_TREHAL

~

Waterextraction:

H2O -> X_H2O

~
