# Supplementary material for: Analysis of optimal phenotypic space using elementary modes as applied to Corynebacterium glutamicum
Source: BMC Bioinformatics. 2006 Oct 12;7:445. doi: 10.1186/1471-2105-7-445 (PMC1617123; doi:10.1186/1471-2105-7-445)
Supplement: Additional File 5 — Demonstration of the methodology using a simple illustrative example. The optimization methodology is applied to an illustrative example to demonstrate the steps used for obtaining the solution. [file 1471-2105-7-445-S5.doc]

**Additional file 5 – Demonstration of the methodology using a simple illustrative example**

The method for the flux assignment of elementary modes can be demonstrated with the help of a simple illustrative example (Figure S1a). A system boundary (dotted line) is considered around all the internal metabolites and the system is closed for this type of metabolites and the fluxes between the internal metabolites are the internal fluxes. But external metabolites are allowed to enter or exit of that theoretical system boundary and exchange flux is the flux by which one external metabolite can enter into the system or one internal metabolite can exit from the system. The biochemical network consists of three internal metabolites (A, B, C) and three external metabolites (X_O_, X_1_, X_2_). There are three exchange fluxes (one of them is reversible) and three internal fluxes (one of them is reversible). The system consists of five elementary modes, which are depicted in Figure S1b, where the extracellular metabolites are connecting from substrate to the products. The fluxes of the external metabolites will be the in terms of fluxes in the elementary modes as given below:

$$\frac{dX_{O}}{dt}=v_{1}+v_{2}+v_{3}+v_{4}$$

$$\frac{dX_{1}}{dt}=4v_{2}+4v_{4}+4v_{5}$$

$$\frac{dX_{2}}{dt}=v_{1}+v_{3}-v_{4}$$

In terms of matrix form, this can be represented as:

$\left[\begin{matrix} 1 & 1 & 1 & 1 & 0 \\ 0 & 4 & 0 & 4 & 4 \\ 1 & 0 & 1 & 0 & -1 \end{matrix}\right]$ $\left[\begin{matrix} v_{1} \\ v_{2} \\ v_{3} \\ v_{4} \\ v_{5} \end{matrix}\right]$ = $\left[\begin{matrix} dX_{O}/dt \\ dX_{1}/dt \\ dX_{2}/dt \end{matrix}\right]$

If the objective function is the maximization of$dX_{2}/dt$, the problem formulation will be as:

Objective function = maximize ($\frac{dX_{2}}{dt}$)

Subject to

$\left[\begin{matrix} 1 & 1 & 1 & 1 & 0 \\ 0 & 4 & 0 & 4 & 4 \end{matrix}\right]$ $\left[\begin{matrix} v_{1} \\ v_{2} \\ v_{3} \\ v_{4} \\ v_{5} \end{matrix}\right]$= $\left[\begin{matrix} dX_{O}/dt \\ dX_{1}/dt \end{matrix}\right]$

and $0\leq v_{i}\leq\infty$ for all $i^{th}$elements

The right hand side of the matrix equations are the measurable quantities (known parameters), while the fluxes of elementary modes ($v_{i}$’s) are the unknowns to be evaluated by means of linear programming.

It can be noted that if two of the accumulation rates of the external metabolites are fixed, the other is automatically fixed due to the overall molar balance. And if only one is provided as input than optimization can yield feasible values of the other two. However, if two are given as inputs, although the third is automatically fixed, the optimization criteria can be used to obtain the fluxes of the individual elementary modes (in this case, there are five elementary modes). And the fluxes of the elementary modes can vary depending on the maximization criteria and will still satisfy the overall molar balance. In the above example, one can maximize or minimize the accumulation rate of the third metabolite to obtain feasible range of fluxes through elementary modes.

The above methodology was used to evaluate the fluxes of elementary modes for the network of *Corynebacterium glutamicum.*


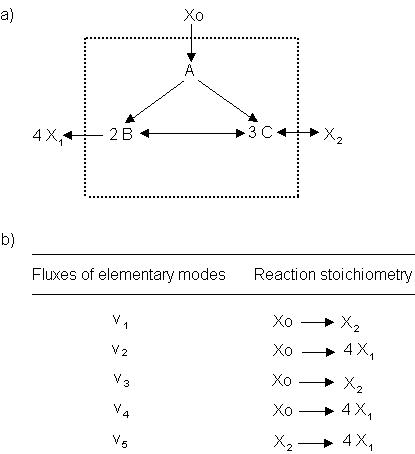


Figure S1. (a) A hypothetical reaction network consisting of three internal metabolites (A, B, C), three external metabolites (X_O_, X_1_, X_2_), three exchange fluxes and three internal fluxes. Double-headed arrows indicate reversible reactions and single headed arrows indicate irreversible reactions. (b) The stoichiometric reactions of elementary modes of the hypothetical reaction network.
